# Focused cardiac ultrasound in preoperative assessment: the perioperative provider’s new stethoscope?

**DOI:** 10.1186/s13741-019-0129-8

**Published:** 2019-11-22

**Authors:** Tara Lenk, John Whittle, Timothy E. Miller, David G. A. Williams, Yuriy S. Bronshteyn

**Affiliations:** 1 Department of Anesthesiology, Mission Hospital, 509 Biltmore Ave, Asheville, NC 28801 USA; 2 Division of General, Vascular, and Transplant, Department of Anesthesiology, Duke University, Durham, NC USA

**Keywords:** Focused cardiac echocardiography, Point of care ultrasound, Preoperative assessment, Perioperative Medicine

## Abstract

Focused cardiac ultrasound (FoCUS)—a simplified, qualitative version of echocardiography—is a well-established tool in the armamentarium of critical care and emergency medicine. This review explores the extent to which FoCUS could also be used to enhance the preoperative physical examination to better utilise resources and identify those who would benefit most from detailed echocardiography prior to surgery. Among the range of pathologies that FoCUS can screen for, the conditions it provides the most utility in the preoperative setting are left ventricular systolic dysfunction (LVSD) and, in certain circumstances, significant aortic stenosis (AS). Thus, FoCUS could help answer two common preoperative diagnostic questions. First, in a patient with high cardiovascular risk who subjectively reports a good functional status, is there evidence of LVSD? Second, does an asymptomatic patient with a systolic murmur have significant aortic stenosis? Importantly, many cardiac pathologies of relevance to perioperative care fall outside the scope of FoCUS, including regional wall motion abnormalities, diastolic dysfunction, left ventricular outflow obstruction, and pulmonary hypertension. Current evidence suggests that after structured training in FoCUS and performance of 20–30 supervised examinations, clinicians can achieve competence in basic cardiac ultrasound image *acquisition*. However, it is not known precisely how many training exams are necessary to achieve competence in FoCUS image *interpretation*. Given the short history of FoCUS use in preoperative evaluation, further research is needed to determine what additional questions FoCUS is suited to answer in the pre-operative setting.

## Background

Preoperative assessment clinics are an essential component of the emerging perioperative care model; they improve care coordination and reduce surgical complications (Blitz et al., 2016; Grocott et al., 2017). Further, the preoperative clinic is an especially valuable setting to complete a thorough preoperative assessment and optimise patients who are at higher risk of perioperative complications, thus permitting increasingly important shared decision-making and individualized care plans. In addition to history, physical, and laboratory tests, costlier and involved investigations are often undertaken, including transthoracic echocardiography (TTE). This is not surprising given the difficulty in predicting the perioperative risk of major adverse cardiac events (MACE) using the 2014 ACC/AHA Guidelines (Fleisher et al., 2014). Provider assessment of patient functional status is often difficult and ambiguous; a recent study showed very poor sensitivity (19%), but high specificity (95%) for subjective assessment of 4 metabolic equivalents confirmed with cardio-pulmonary exercise testing (CPET) (Wijeysundera et al., 2018). Given the common clinical question of exercise capacity, it is not surprising that many TTEs that are ordered are found to be normal even in high-risk patients. In a retrospective study of 570 high-risk patients who underwent TTE prior to non-cardiac surgery, approximately 50% were found to have essentially normal echocardiograms (Rohde et al., 2001). In this study, clinical characteristics most associated with an abnormal TTE were age greater than 70, history of congestive heart failure (CHF) or myocardial infarction (MI), systolic murmur, and/or evidence of left ventricular hypertrophy (LVH) on electrocardiogram (Rohde et al., 2001).

In pursuit of a cost-effective, bedside test that provides objective insight into cardiac function, focused cardiac ultrasound (FoCUS) presents an intriguing possibility in the preoperative setting. FoCUS can enhance the physical examination during the clinic visit, which may help better steward resources and identify those patients who would most benefit from detailed echocardiography. However, FoCUS also has important limitations that distinguish and currently prevent it from becoming a substitute for comprehensive echocardiography in many situations.

### Focused cardiac ultrasound and transthoracic echocardiography

Focused cardiac ultrasound (FoCUS) is defined by the American Society of Echocardiography (ASE) as a *qualitative* transthoracic ultrasound examination of the heart performed and interpreted at the bedside by clinicians who have at least focused training in cardiac ultrasound image acquisition and interpretation (Spencer et al., 2013). FoCUS typically makes use of simple ultrasound equipment and basic ultrasound modes: primarily B-mode (2D/greyscale) and occasionally colour Doppler. In contrast, transthoracic echocardiography is a *quantitative* ultrasound exam of the heart employing both simple (B-mode, colour Doppler) and advanced modalities (e.g. spectral Doppler, strain imaging, 3D modes). TTE images are usually obtained by a sonographer at the bedside and then separately interpreted by a physician with comprehensive training in both (i) cardiac ultrasound pathology and (ii) advanced ultrasound imaging modalities (see Table 1) (Via et al., 2014). In contrast to the comprehensive scope of practice of TTE, the FoCUS scope of practice has been defined narrowly to include the following (Via et al., 2014):

- Qualitative assessment of left ventricular size and systolic function
- Qualitative assessment of right ventricular size and systolic function
- Identifying extremes of volume status
- Identifying the presence vs. absence of pericardial effusions
- Identifying gross intra-cardiac masses
- Identifying gross signs of chronic heart disease
- Identifying morphologic (2D) clues of gross valvular disease

**Table 1:** Key differences between focused cardiac ultrasound (FoCUS) and transthoracic echocardiography (TTE)

Focused cardiac ultrasound (FoCUS)	Transthoracic chocardiography (TTE)
Qualitative ultrasound assessment of the heart	Quantitative ultrasound assessment of the heart
Requires practitioners to be familiar with a limited number of transthoracic cardiac views, a limited number of pathologies, and simple imaging modes (B-mode ± colour Doppler)	Requires practitioners to have comprehensive training of all transthoracic cardiac views, ultrasound imaging modes, and ultrasound-relevant cardiac pathologies
Can be performed using simple equipment	Requires advanced equipment
Always goal-directed	Can be goal-directed (limited) or comprehensive
Always performed and interpreted at the point-of-care	Usually performed at the bedside and interpreted separately

FoCUS is a goal-directed examination intended to answer time-sensitive clinical questions. Its results may influence the bedside clinician to do either, both, or none of the following: (i) change the patient’s treatment plan; and/or (ii) order another diagnostic test (e.g. TTE) (Beaulieu, 2007). Although FoCUS is well-established in the armamentarium of critical care and emergency medicine (Levitov et al., 2016; Ultrasound Guidelines, 2017), it remains unclear what role FoCUS has in the preoperative evaluation of patients. Can FoCUS evolve echocardiography from the cardiology laboratory to the preoperative clinic, increasing convenience and access for patients (Royse et al., 2012)? Can FoCUS further help preoperative clinics—and thus healthcare systems—decrease expensive and often unnecessary TTEs while improving risk stratification through a significantly enhanced physical examination (Terkawi et al., 2013)?

### The value of preoperative FoCUS

There is observational evidence that preoperative FoCUS can change and may even improve the clinical management of patients. One small study with 49 patients found that FoCUS changed perioperative management in 84% of cases. Within the 50% of cases that had a systolic murmur, 2 important pathologies were identified: 19 cases of aortic stenosis (8 cases being moderate or severe), and 10 cases of poor LV function (Cowie, 2009). A recent systematic review identified three anaesthesia-related studies where preoperative FoCUS was used before emergency surgery in those who had a clinical indication for FoCUS. FoCUS changed the clinical diagnosis 51–67% of the time, with left ventricular dysfunction and new valvular disease being the most common new diagnoses (Heiberg et al., 2016). In a separate retrospective case series, a hospital analysed the impact of a perioperative FoCUS consulting service: of the 170 examinations the consulting service performed, 75% were performed in the preoperative period. Of these, 82% changed patient management. Referral for TTE was observed in 34% as a result of the FoCUS examination. When a TTE was carried out as a consequence of FoCUS, 91% of the examinations resulted in pathological findings, a testament to the potential of FoCUS to steward healthcare resources appropriately (Cowie, 2011).

In the specific setting of the preoperative assessment clinic, Canty et al. studied the impact of preoperative FoCUS on a cohort of 100 high-risk patients scheduled for a variety of surgeries (Levitov et al., 2016). FoCUS was performed by an anaesthetist experienced in echocardiography, and all images were later reviewed by a cardiologist to confirm adequate interpretation. In this cohort of patients who were recognized to have an indication for FoCUS (56% due to suspected valvular disease), hemodynamically significant cardiac disease was found in 34% of patients, and no concerning ultrasound findings in the other 76%, resulting in a step-down in clinical anaesthetic resources in 49% of cases. When questioned, anaesthetists assigned to the preassessment clinic who were not aware of the results of FoCUS examinations indicated that they would have requested a TTE in 84% of the subjects enrolled in this study. This further hints at the potential of FoCUS to improve triaging of healthcare resources (Canty et al., 2012).

In the USA, in addition to better stewardship of healthcare resources, preoperative FoCUS may help healthcare systems in another less immediately obvious way by improving the accuracy of each institutions’ case mix index. For example, when preoperative FoCUS detects and documents severe left ventricular systolic dysfunction, hospital reimbursement for subsequent surgery has the potential to increase (Zimmerman, 2018). Therefore, when adequately trained providers perform preoperative FoCUS, it can help ensure that hospitals are appropriately reimbursed for the complexity of perioperative care that they deliver.

### How many tests are needed to become proficient?

Given the critical importance of diagnostic accuracy when performing FoCUS, a logical next question is how much training is required to become proficient in this skillset? Cardiac ultrasound interpretation is inherently subjective and operator-dependent, such that even senior echocardiographers routinely disagree with one another and even with themselves when visually assessing pathologies such as left ventricular regional wall motion abnormalities (Blondheim et al., 2010). Given the difficulty that expert echo-cardiologists have interpreting TTE, many healthcare systems are understandably reluctant to permit clinical decision-making based solely on FoCUS examination findings. To maximize patient safety, providers seeking to perform FoCUS should first receive adequate training and demonstrate competency in this skillset. Unfortunately, there exist many ambiguities regarding credentialing and privileging for point-of-care ultrasound generally and FoCUS specifically (Kimura, 2017). Existing well-developed guidelines for competency in other realms of echocardiography, including perioperative transesophageal echocardiography (TEE), provide a stark contrast to the lack of guidelines for FoCUS. This ambiguity makes it difficult for many to adopt FoCUS comfortably into their practice (Alfirevic, 2015).

The most stringent recommendations for hand-carried ultrasound usage come from professional cardiology and echocardiography organizations, which recommend achieving at least level 1 competence in order to independently perform and interpret hand-carried ultrasound. Training for level 1 includes performing 75 examinations, interpreting 150, and completing 3 months of didactic learning (Beaulieu, 2007). However, these competency requirements have been designed with the cardiovascular specialist in mind and thus are geared toward quantitative echocardiography rather than the use FoCUS to help answer qualitative questions. Other specialties have released less stringent requirements, e.g., emergency medicine has suggested 25–50 exams are adequate to achieve competency in FoCUS (Ultrasound Guidelines, 2017). In the critical care arena, a recent publication described basic competency as the ability to achieve high-quality images on all standard views, ability to distinguish normal vs. abnormal and seek appropriate referrals after completing 50–100 exams (Price et al., 2008). The British Society of Echocardiography calls for 10 supervised exams and 50 individually acquired exams with interpretation overseen by a mentor in order to become accredited in focused intensive care echocardiography (FICE) (Echocardiography BSo, n.d.). In contrast, a different expert consensus statement in critical care concluded the following: (i) 30 fully supervised FoCUS exams may be the minimum required to achieve competence in image acquisition; (ii) the minimum number of studies required to achieve competence in image interpretation should be based on teacher/supervisor determination (International expert statement on training standards for critical care ultrasonography, 2011). Lending support to this latter consensus statement, Millington et al. studied the learning curve of FoCUS image acquisition and image interpretation skills among residents from multiple acute care specialties (Millington et al., 2017). Prior to the start of the study, the learners performed a median of 8 training exams and received extensive didactic preparation consisting of 2 days of lectures and a month-long elective in echocardiography. The authors found that competence in image acquisition plateaued at around 20 exams, but competence in image interpretation required a larger volume of studies (Millington et al., 2017). Based on these studies, a preoperative provider new to cardiac ultrasound would likely need to undergo a period of didactic training and perform at least 20–30 supervised exams, in order to acquire images competently. Further, this individual would need additional oversight by a more experienced practitioner for an unknown period of time until competency in image interpretation is demonstrated. Notably, this amount of training would only qualify the provider to perform and interpret FoCUS, not TTE. It thus becomes useful to understand the potential pathologies not seen with FoCUS that are relevant to the perioperative care of patients.

### Does FoCUS have blind spots?

One valid concern about FoCUS is that it will miss clinically significant findings unrelated to the narrow goals of the examination. Although publications on this potential shortcoming of FoCUS are scarce, some inferences can be drawn from related studies about *limited* TTE. Kimura et al. looked at a series of 172 *comprehensive* TTEs performed on high-risk patients for one of two indications: “rule out pericardial effusion” or “source of embolus”. The authors then constructed a focused (limited) protocol for each of these two indications and re-examined the comprehensive TTE images by only looking at ultrasound clips allowed by the focused protocol. In the “rule-out pericardial effusion” group and those under 65 years old, a focused TTE would have missed incidental diagnoses only 3% of the time. Incidental findings were more commonly found in the older group with more comorbidity (46%). The most frequently missed findings were valve disease and LV dysfunction (Kimura et al., 1998). In a different study of 43 patients with a history of hypertension who had a comprehensive TTE performed to evaluate for LV mass, only two (5%) would have had missed findings (bicuspid aortic valve and multiple hepatic cysts) had their TTE been goal-oriented rather than comprehensive (Shub et al., 1995). Although these studies do suggest a low rate of false negatives with a limited TTE, these false negatives are likely to increase with the application of *FoCUS* since the FoCUS exam can be performed by providers with only basic training in cardiac ultrasound.

In other words, operator experience influences the diagnostic accuracy of FoCUS. For instance, two large studies (each with a sample size > 500) showed that experienced echocardiographers performing FoCUS using simple, hand-held equipment could identify important findings with a sensitivity of 97% and specificity of 93% (Galasko et al., 2006; Galasko et al., 2003). As the experience level of the echocardiographer decreases, in general, sensitivity drops but specificity remains acceptable. In a similar study, an internist with 20 h of didactic learning and 40 prior examinations performed FoCUS in a minority health clinic on 43 patients who also received a comprehensive TTE for validation. The internist’s sensitivity and specificity were variable: both above 95% for regurgitant valvular lesions using colour Doppler, but 40% and 100%, respectively, for ventricular dysfunction < 40% (Kirkpatrick et al., 2004). The high specificity could potentially be useful as a rule-in strategy decrease TTE ordering. However, this study also strongly highlights the need for expert oversight during the early part of the FoCUS learning curve due to the internist’s low sensitivity. Decara et al. compared expert echocardiographers with internal medicine residents who had undergone 20 h of didactic learning and 20 prior examinations in 300 high-risk patients who had otherwise been referred for TTE. Trainees and experts were both best able to identify left ventricular systolic dysfunction (LVSD): the miss rate was only 3–4% in both groups. However, both groups missed right ventricular (RV) dysfunction or regional motion wall abnormalities 25–45% of the time. For clinically important findings, the residents had a sensitivity of 80% and specificity of 97% (DeCara et al., 2003). Overall, sensitivity continually improves with experience, while specificity seems to be achieved early. This suggests that an appropriate level of experience, however still undefined, is needed in order to decrease the number of false negatives, as the number of false positives is already low even in inexperienced operators.

Combining these findings with the accepted scope of practice of FoCUS helps illuminate both FoCUS’ strengths and shortfalls in augmenting the preoperative evaluation. First, the ASE-accepted scope of practice of FoCUS excludes the use of spectral Doppler and quantitative methods, which means that diastolic function and quantification of either valvular disease or pulmonary hypertension cannot be comprehensively assessed with FoCUS. Second, assessment for dynamic left ventricular outflow tract (LVOT) obstruction (or risk thereof) routinely requires spectral Doppler and provocative manoeuvres that fall outside the scope of FoCUS. Third, regional wall motion assessment is an advanced skillset that falls outside the scope of practice of FoCUS (Via et al., 2014). Although RV systolic function assessment is included in the scope of practice of FoCUS as defined by the ASE, assessing RV function is a challenging and subjective skillset, and the minimum number of training studies required to achieve competence is unknown (Via et al., 2014). In contrast, global left ventricular (LV) systolic function assessment is perhaps the most useful application of preoperative FoCUS: this skillset seems to be easily learned and can help identify patients with LVSD, a condition with high perioperative risk (Spencer et al., 2013). Further, FoCUS may also allow qualitative screening for gross aortic stenosis in certain situations.

### Indications for preoperative FoCUS

Although there exist no published guidelines identifying indications for preoperative FoCUS, several professional medical societies have published guidelines identifying indications for preoperative TTE. Of relevance to FoCUS, these guidelines, to varying degrees, support the use of TTE in the following situations: (i) documented ischemic heart disease with reduced functional capacity (< 4 METS); (ii) unexplained dyspnoea or poor functional status; and (iii) finding of a murmur, especially in symptomatic patients (Fleisher et al., 2014; Indications for Echocardiography: British Society of Echocardiography, 2011; Douglas et al., 2011). In the absence of evidence to the contrary, it might seem reasonable to use these same indications to justify performing preoperative FoCUS in order to enhance the physical examination and clinical decision-making. However, since FoCUS has a much more limited scope of practice than TTE, clinicians must be aware that a preoperative FoCUS exam will only identify a subset of possible pathologies. Among the range of pathologies that FoCUS can potentially identify, the most pertinent conditions in the preoperative setting are left ventricular systolic dysfunction (LVSD) and, in certain circumstances, significant aortic stenosis.

### Using FoCUS for suspected left ventricular systolic dysfunction

Of all of the potential applications of FoCUS in the preoperative clinic, assessment for suspected left ventricular systolic dysfunction (LVSD) is the most promising. LVSD is a clinical entity that is identifiable, somewhat prevalent, frequently missed by physical examination, associated with significant perioperative morbidity and mortality, and treatable with appropriate therapy (Spencer et al., 2013). Prevalence of LVSD can vary greatly, from 2–5% seen in a large community sample (Spencer et al., 2013), up to 30% seen in a large sample of 10,710 symptomatic patients suspected of having LVSD (Madhok et al., 2008). Further, the perioperative risk burden is high. In a prospective cohort study of 1005 vascular surgery patients in which 20% had asymptomatic LVSD, and 20% had known heart failure (confirmed by echocardiography), the odds ratio of 30-day CV events (myocardial ischemia, infarction, and 30-day cardiovascular mortality) was 2.3 and 6.8, respectively (Flu et al., 2010). Unfortunately, LVSD is notoriously difficult to convincingly identify using traditional clinical and physical exam findings alone without using echocardiography. A meta-analysis showed that no clinical or physical exam findings were sufficient to “rule-in” or “rule-out LVSD, highlighting the potential utility of FoCUS to improve patient assessment (Madhok et al., 2008). Of all of the clinical entities that FoCUS can identify, LVSD is the most readily learned: medical residents who were blinded to all other clinical variables were able to achieve a sensitivity and specificity of ~ 94% for an ejection fraction less than 40% after 20 practice exams (Razi et al., 2011).

Although FoCUS can identify LVSD with relative ease, the value of this in the pre-operative evaluation is not straightforward. In a patient with poor functional status, a FoCUS exam showing LVSD would still necessitate a comprehensive echocardiogram to evaluate for other ancillary problems that fall outside the scope of FoCUS (e.g. pulmonary hypertension, diastolic dysfunction). Conversely, if the FoCUS findings on the same patient showed normal LV systolic function, a comprehensive echocardiogram would still be indicated to pursue other cardiac causes of functional impairment. In sum, a patient with poor functional status would likely require a TTE regardless of the findings of the FoCUS exam. However, in patients at high risk of cardiovascular disease who report a robust functional status (> 4 METS), current guidelines would not support echocardiography. Thus, in this patient population, FoCUS has the potential to detect asymptomatic LVSD. In so doing, FoCUS can help perioperative providers identify this dangerous and often silent condition, potentially allowing more accurate risk stratification and more targeted patient care.

### What about systolic murmurs?

A new, uncharacterized systolic murmur is a common clinical conundrum in the preoperative clinic. Of the many possible diagnoses, aortic stenosis (AS) is the most ominous. Unfortunately, the intensity of aortic systolic ejection murmur does not help identify the severity of AS (Aronow & Kronzon, 1987; Das et al., 2000). Further, perioperative morbidity is high in patients with AS. A retrospective study using 4:1 propensity matching found double the 30-day mortality rate in AS patients than controls (2.1 vs. 1%), and a three-fold increase in post-op MI (3 vs. 1.1%) (Agarwal et al., 2013). Similarly, in a study of 4300 patients (of which 570 had a preoperative TTE), an aortic valve peak instantaneous gradient greater than 40 mmHg had the highest odds ratio of all echocardiographic variables at 6.3 (1.3–31 95%) for perioperative cardiac complications (Rohde et al., 2001). Given the danger of this clinical entity, it might seem reasonable to obtain a preoperative FoCUS in patients presenting with a new systolic murmur, especially if the patient is symptomatic.

However, in practice, it is much more challenging to use FoCUS to screen for aortic stenosis than to screen for LVSD. Colour and spectral Doppler technology can quantify the severity of valvular disease, but require advanced training beyond the scope of FoCUS (Spencer et al., 2013). In a subset of cases, the FoCUS practitioner may be able to clearly visualize a well-opening aortic valve. This simple visual finding effectively rules out significant aortic stenosis. However, studies on the competency of FoCUS with aortic stenosis are lacking, and thus clinicians should have a very low threshold to order a TTE when any abnormality of the aortic valve is noticed or images are at all inadequate. Further, in symptomatic patients, the pre-test probability of aortic stenosis may be so high that even a seemingly negative FoCUS exam may still warrant pursuit of a TTE to evaluate for AS and/or other potential cardiac causes of lifestyle limitation. Thus, in *symptomatic* patients with a systolic murmur, a FoCUS may not be useful because the patient will end up receiving a TTE regardless of the FoCUS findings. In contrast, FoCUS may thus have the most value in *asymptomatic* patients with a systolic murmur. A negative FoCUS exam may spare them what would have been an unnecessary TTE. Conversely, a positive exam could pick up those patients who are still asymptomatic but have an underlying serious disease. FoCUS images of a heavily calcified and poorly opening aortic valve in an otherwise asymptomatic patient would lead the clinician to order a well-deserved comprehensive TTE to further characterize their disease.

## Future directions

Does FoCUS have value in the pre-operative evaluation beyond two important yet narrow indications (i.e. screening for LVSD and AS in a subset of patients)? Can FoCUS broaden its relevance to the pre-operative evaluation? Arguably, one of two things must happen for FoCUS to become comprehensively useful in preoperative screening: the FoCUS exam itself, or ultrasound technology must progress beyond their current state. Fortunately, these outcomes are plausible. First, as ultrasound becomes more intricately woven into medical schools and training programs and the skills of providers continue to evolve in this arena, evidence-based guidelines might emerge that broaden the scope of practice of FoCUS to include a broader list of cardiac pathologies. Second, the growing sophistication of machine learning algorithms in diagnostic imaging may eventually enable automated interpretation of FoCUS exam images at the bedside for a broad range of cardiac conditions (Alsharqi et al., 2018). Until either of these events occurs, clinicians will likely need to rely on currently available tools for cardiovascular evaluation and risk stratification (e.g. history/physical, brain natriuretic peptide levels, CPET), while utilizing FOCUS for its current indications: to look for LVSD or AS in a subset of patients.

## Conclusions

FoCUS is currently a limited but still potentially useful tool in the preoperative setting and may gain a stronger footing and a broader scope in the near future to enhance the cardiac physical examination and better steward additional cardiac testing. When an experienced operator performs FoCUS during the clinic visit, the finding of grossly normal biventricular systolic function and a well-opening aortic valve effectively rules out several potentially life-threatening conditions of high relevance to perioperative providers. Conversely, the finding of significant impairment of the function of either ventricle and/or minimal opening of the aortic valve should prompt an escalation of testing and monitoring and/or re-evaluation of the patient’s suitability for surgery. However, FoCUS neither quantifies the severity of any kind of valvular disease nor does it assess for multiple other conditions of relevance to perioperative care, e.g., pulmonary hypertension, LVOT obstruction, and diastolic dysfunction. These blind spots effectively limit the relevance of FoCUS in the preoperative evaluation of symptomatic patients: regardless of whether a FoCUS exam is positive or negative for LVSD or AS, other important causes of functional limitation may be missed, thus prompting the need for a TTE seemingly regardless of the FoCUS findings. Fortunately, these limitations need not be permanent. Either the expansion of the scope of practice of FoCUS or continued advancement in automated image interpretation or both could render this modality especially useful in preoperative screening in the not-too-distant future. Until then, as high-risk clinics continue to emerge and contribute to population health, screening for LVSD or AS in high-risk pre-selected populations could be its own valuable and relatively simple risk stratification tool.

## Data Availability

Not applicable

## Abbreviations

AS: Aortic stenosis
ASE: American Society of Echocardiography
CHF: Congestive heart failure
CPET: Cardiopulmonary exercise testing
FICE: Focused intensive care echocardiography
FoCUS: Focused cardiac ultrasound
LV: Left ventricle
LVH: Left ventricular hypertrophy
LVOT: Left ventricular outflow tract
LVSD: Left ventricular systolic dysfunction
MACE: Major adverse cardiac events
METs: Metabolic equivalents
MI: Myocardial infarction
TEE: Transesophageal echocardiography
TTE: Transthoracic echocardiogram
